# Ferroelectric Engineered Electrode‐Composite Polymer Electrolyte Interfaces for All‐Solid‐State Sodium Metal Battery

**DOI:** 10.1002/advs.202105849

**Published:** 2022-03-06

**Authors:** Yumei Wang, Zhongting Wang, Feng Zheng, Jianguo Sun, Jin An Sam Oh, Tian Wu, Gongxuan Chen, Qing Huang, Masashi Kotobuki, Kaiyang Zeng, Li Lu

**Affiliations:** ^1^ National University of Singapore (Chongqing) Research Institute Chongqing 401123 P.R. China; ^2^ Department of Mechanical Engineering National University of Singapore 9 Engineering Drive 1 Singapore 117575 Singapore; ^3^ College of Materials Science and Engineering Chongqing University Chongqing 400044 P.R. China; ^4^ Institute of Materials Research and Engineering Hubei University of Education Wuhan 430205 P. R. China; ^5^ Battery Research Center of Green Energy Ming Chi University of Technology 84 Gungjuan Rd., Taishan Dist. New Taipei City 24301 Taiwan; ^6^ National University of Singapore (Suzhou) Research Institute Suzhou 215125 P.R. China

**Keywords:** cyclic performance, electrolyte‐electrode interfaces, ferroelectric engineering, solid‐state batteries, stability

## Abstract

To enhance the compatibility between the polymer‐based electrolytes and electrodes, and promote the interfacial ion conduction, a novel approach to engineer the interfaces between all‐solid‐state composite polymer electrolyte and electrodes using thin layers of ferroelectrics is introduced. The well‐designed and ferroelectric‐engineered composite polymer electrolyte demonstrates an attractive ionic conductivity of 7.9 × 10^–5^ S cm^–1^ at room temperature. Furthermore, the ferroelectric engineering is able to effectively suppress the growth of solid electrolyte interphase (SEI) at the interface between polymer electrolytes and Na metal electrodes, and it can also enhance the ion diffusion across the electrolyte‐ferroelectric‐cathode/anode interfaces. Notably, an extraordinarily high discharge capacity of 160.3 mAh g^–1^, with 97.4% in retention, is achieved in the ferroelectric‐engineered all‐solid‐state Na metal cell after 165 cycles at room temperature. Moreover, outstanding stability is demonstrated that a high discharge capacity retention of 86.0% is achieved over 180 full charge/discharge cycles, even though the cell has been aged for 2 months. This work provides new insights in enhancing the long‐cyclability and stability of solid‐state rechargeable batteries.

## Graphical abstract

The bottleneck limiting the cyclic performance and long‐term stability of solid‐state batteries has changed from the ion conduction of electrolyte itself to electrolyte‐electrode interfaces. Herein, this issue is addressed via ferroelectric engineering at the electrolyte‐electrode interfaces. A high reversible capacity of 160.3 mAh g^−1^ with a retention of 97.4% is demonstrated in this all‐solid‐state Na metal cell after 165 cycles at room temperature.

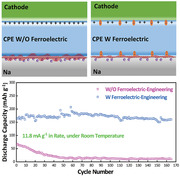

## 1 Introduction

Developing high‐capacity, long‐cyclability, and cost‐effective solid‐state sodium batteries are appealing for electric vehicles and large‐scale energy storage systems.^[^ ^1^ ^]^ The non/less flammable solid‐state electrolytes are believed to enhance the battery safety, especially towards the very reactive metal anodes (e.g., Li, Na, and K). As an important component, solid‐state electrolytes should be electrochemically and chemically stable with both cathodes and anodes and have high ionic but negligible electronic conductivity over the battery operation voltage and temperature range.^[^ ^2^ ^]^ Inorganic solid electrolytes possess the advantages of fast ion conduction and good electrochemical and chemical stability with respect to electrodes. Nevertheless, the poor interfacial contact between these rigid electrolytes and the solid electrodes hinders their wide applications. On the contrary, polymer electrolytes (PEs) and composite polymer electrolytes (CPEs) are highly flexible, and thus they could form intimate contact with electrodes.^[^ ^3^ ^]^ However, the unsatisfied ionic conductivity limits the operation of most PEs/CPEs at room temperature.^[^ ^4^, ^5^, ^6^, ^7^, ^8^, ^9^, ^10^, ^11^, ^12^, ^13^, ^14^, ^15^, ^16^ ^]^ Although highly improved ionic conductivities have recently been reported in some well‐designed CPEs, solid‐state Na metal cells using such CPEs still show quick capacity fading during cycling, especially at room temperature.^[^ ^17^, ^18^ ^]^ It is believed that the poor electrolyte‐electrode compatibility and the low ion‐conducting interfaces are the main causes that degrade the battery performances, even though there exists good physical contact.^[^ ^19^ ^]^

**Figure 1:**
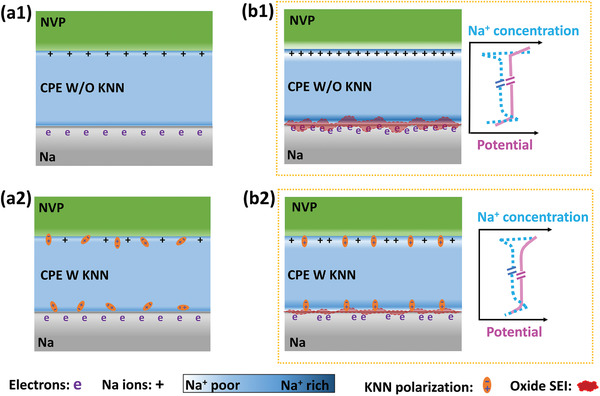
Schematic illustrations of space‐charge build‐up in the all‐solid‐state NVP | CPE | Na cells and SEI formation: a1) without interfacial engineering and before charging, b1) without engineering and after charging, a2) with KNN‐interfacial engineering and before charging, b2) with KNN‐interfacial engineering and after charging.

It is commonly accepted that the sluggish ion migration across the electrode‐electrolyte interfaces is caused by the interfacial reactions and space‐charge layers.^[^ ^20^, ^21^, ^22^, ^23^, ^24^, ^25^ ^]^ Space‐charge layers form when two materials with different chemical/electrical potentials are brought into contact, and the mobile charges will be redistributed under the driving force of potential differences. As illustrated in the concept of the all‐solid‐state Na metal battery (**Figure** **1**(a1)), some Na^+^ ions will transport through the interface between the CPE and the cathode (such as Na_3_V_1.85_Fe_0.15_(PO_4_)_3_ (NVP)) under the driving force of interfacial potential differences, until equilibrium reached, leaving a Na^+^‐accumulated layer at the interfacial CPE and a nearby Na^+^‐deficient layer at the interfacial cathode. Consequently, the ion‐concentration change at the cathode‐CPE interface would increase the energy barrier for Na^+^ migration, thus leading to large interfacial resistance.^[^ ^19^, ^22^, ^24^ ^]^ At the anode side, the interfacial potential drop causes a similar Na^+^‐redistribution at the interfacial CPE‐anode. Besides, an electron‐accumulated region forms at the interfacial anode, to maintain the electrical neutrality within the interfacial anode. During charging, more mobile Na^+^ will be blocked as the cathode potential increases, and the interfacial resistances are therefore exacerbated (Figure 1(b1)).^[^ ^25^ ^]^ On the other hand, the cathodic stability of the poly(ethylene oxide) (PEO, widely used in solid‐state batteries) based polymer electrolytes has long been neglected. When encountering a very reducing metal, such as Li and Na, the PEO‐based electrolytes could react with it.^[^ ^26^, ^27^ ^]^ Oxide SEI appears as the reaction product and further resists the Na^+^ conduction at the electrolyte‐anode interface. The SEI grows even thicker and becomes more resistant against Na^+^ transportation during battery cycling,^[^ ^18^ ^]^ since the accumulated electrons at the interfacial anode would continuously combine with the mobile Na^+^ and transform into new Na to further react with the PEO‐based electrolytes (Figure 1(b1)).

To date, the bottleneck limiting the development of solid‐state rechargeable batteries has changed from the ion conduction of electrolyte itself to electrolyte‐electrode interfaces. However, this critical problem has yet been well understood and solved. Although several strategies have been taken to improve the interfaces between the inorganic solid electrolytes and cathodes,^[^ ^20^, ^21^, ^28^, ^29^, ^30^ ^]^ severe issues between the PEs/CPEs and electrodes, especially the highly reactive alkali metal anodes, have never been studied. In this work, we improve the CPE‐electrode interfaces in the all‐solid‐state Na metal cell through ferroelectric engineering (ferroelectric KNN, K_0.5_Na_0.5_NbO_3_, as shown in Figure 1(a2,b2)). Fundamentally during charging, the KNN polarizations could be switched under the interfacial potential drop. Then the aligned KNN polarizations would build a local electric field to weaken the potential gaps at the electrolyte‐ferroelectric‐cathode/anode interfaces and attenuate the interfacial space charges. Consequently, the interfacial ion conduction would be facilitated in the reduced space‐charge regions, and the less accumulated electrons at interfacial anode could refrain the further growth of SEI and decrease the SEI‐induced interfacial resistance. In the following discharge process, the polarized KNN domains would not totally rotate back, although their polarization might decrease to some extent. The interfacial ion migration could still be promoted via the less‐oriented KNN polarizations. According to our operando electrochemical impedance spectroscopy analysis, greatly reduced interfacial resistance was maintained in the KNN‐engineered cells, compared with the non‐engineered cell, during the long‐term charge process. An extremely high discharge capacity of 160.3 mAh g^–1^, with 97.4% in retention, was achieved in the engineered cell after 165 cycles at room temperature. Moreover, superior capacity retention of 86.0% was achieved in the engineered cell over 180 full charge/discharge cycles, even though the cell had been suspended for 2 months during cycling.

## 2 Results and Discussion

**Figure 2:**
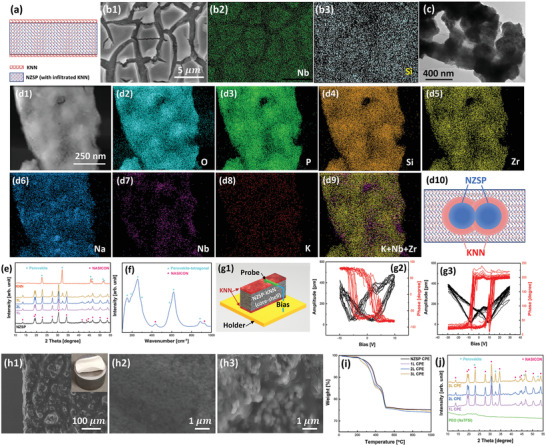
a) Schematic illustration of KNN‐coated NZSP framework; b1) surface morphology, b2) Nb mapping and b3) Si mapping of KNN‐NZSP frameworks; c) STEM image; d1) HAADF image, d2) O mapping, d3) P mapping, d4) Si mapping, d5) Zr mapping, d6) Na mapping, d7) Nb mapping, d8) K mapping, and d9) K+Nb+Zr mapping of the KNN‐NZSP framework, d10) schematic drawing of the core (NZSP)‐shell (KNN) structure inside the KNN‐NZSP framework; e) XRD patterns of the KNN‐NZSP frameworks in comparison with the KNN and NZSP control samples; f) Raman spectroscopy of the KNN‐NZSP 2L framework; g1) schematic illustration of the PFM measurement, g2) local PFM hysteresis loops (10 cycles) of KNN‐NZSP 1L and g3) KNN‐NZSP 3L; h1) low‐magnification cross‐section, h2) high‐magnification of surface, h3) cross‐section morphologies of the KNN‐NZSP CPE‐3L (inset of (h1) is the photograph of the flexible CPE‐3L); i) TGA of the NZSP CPE and KNN‐NZSP CPEs; j) XRD patterns of the KNN‐NZSP CPEs in comparison with the PEO (NaTFSI) control sample.

To easily switch the direction of ferroelectric polarizations, the thickness of the KNN layer should be designed as thin as possible, which makes the interfacial KNN difficult to be free‐standing. In addition, the porous KNN structure can further reduce the pinning effect of the ferroelectric domain walls. As shown in **Figure** **2**a,b, the thin islands of KNN are coated on the surfaces of NZSP (Na_3_Zr_2_Si_2_PO_12_, the famous Na‐ion super‐conductor) ceramic frameworks. Inside the coated framework, the ceramic skeleton is 3D inter‐connected with nano‐size pores (Figure 2c). During coating, KNN not only grew on the outer surfaces of NZSP framework but infiltrated into the framework as a thin shell on the core of NZSP grain (Figure 2d and Figure S1, Supporting Information). This core‐shell structure would not degrade the conductivity or the electrochemical stability of the CPEs (will be present in the following section). Three KNN‐NZSP frameworks with different KNN coating layers, labeled as 1L, 2L, and 3L, respectively were prepared. The XRD patterns of the 1L, 2L, and 3L frameworks only show characteristic peaks of NASICON‐structured NZSP and perovskite KNN (Figure 2e). No impurity phases are observed, indicating the KNN coatings did not affect the crystalline structure of NZSP. Raman spectroscopy further reveals the NbO_6_ vibration in the tetragonal perovskite KNN (Figure 2f). The ferroelectricity of the KNN coatings was investigated by the piezoresponse force microscopy (PFM) through the converse piezoelectric effect.^[^ ^31^ ^]^ Stable ferroelectric switching is demonstrated in the KNN‐NZSP frameworks (Figure 2g), that is obvious polarization switching in the 1L sample under the bias of ≈3.0 V, and under ≈8.0V in the 3L sample because of the increased thickness of KNN coatings. As the PFM characterization is conducted under a tip bias over nanoscale, the measured switching voltages are often overestimated. In fact, the switch of KNN polarizations could be easily triggered under the interfacial potential drop during battery charging, and no additional complicated poling process is needed.

After preparing the porous KNN‐NZSP ceramic frameworks, highly flexible CPEs were fabricated by fully filling the pores of the frameworks with the melted PEO (NaTFSI) polymer electrolyte (Figure 2h). As observed on the surface of KNN‐NZSP CPEs (Figure 2(h2)), KNN particles are well wrapped with the polymer, and the polymer wrappings provide the necessary conduction pathways for Na^+^. Inside the KNN‐NZSP CPEs (Figure 2(h3)), the ceramic particles are also well‐covered by the polymer and integrated with sufficient polymer bindings, forming 3D inter‐connected routes for Na^+^ migration. Figure 2i reveals the gravimetric changes of both NZSP CPE and KNN‐NZSP CPEs, showing their good thermal stability till 200 °C, above which the polymer begins to thermally decompose. The weight fraction of polymer fillers in all CPEs are similar, approximately 25 wt.%. Figure 2j reveals the crystalline structure of the KNN‐NZSP CPEs, clearly showing the characteristic diffraction peaks from PEO (NaTFSI), perovskite KNN and NASICON‐type NZSP. No other diffraction peaks are observed.

**Figure 3:**
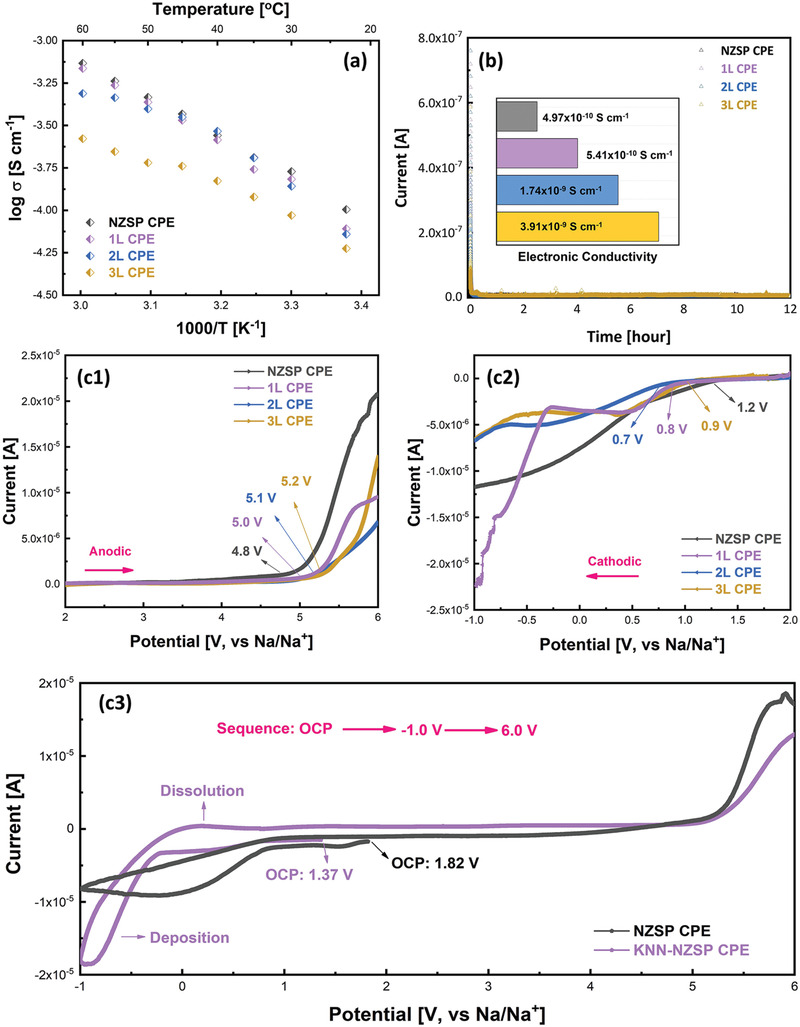
a) Temperature‐dependent ionic conductivity of the NZSP CPE and KNN‐NZSP CPEs, b) potentiostatic polarization curves of the NZSP CPE and KNN‐NZSP CPEs (tested at room temperature under 0.5 V; Insets are the calculated electronic conductivity); c1) anodic and c2) cathodic linear sweep voltammetry curves of the NZSP CPE and KNN‐NZSP CPEs (measured at room temperature with the rate of 0.5 mV s^–1^), c3) current‐potential curves of the NZSP CPE and KNN‐NZSP CPE‐1L (measured at room temperature with a scanning rate of 0.5 mV s^–1^).

Ionic conductivity is an utmost important factor for electrolytes. To investigate the effect of the ferroelectric coatings on the conductivity of CPEs, the temperature dependent ionic conductivity for the non‐engineered and KNN‐engineered CPEs were measured (**Figure** **3**a and Figure S2, Supporting Information). Although KNN is not a Na^+^‐conductor, the polymer wrappings outside the KNN/KNN‐NZSP ceramic particles provide continuous pathways for the Na^+^ migration. As such, high ionic conductivity of 1.0 × 10^–4^ S cm^–1^ at room temperature and 7.2 × 10^–4^ S cm^–1^ at 60 °C have been achieved for the non‐engineered NZSP CPE, whereas the ionic conductivity of the KNN‐engineered CPEs decreases with the thickness of KNN layers. At room temperature, the ionic conductivity decreases to 7.9 × 10^–5^ S cm^–1^ for the KNN‐NZSP CPE‐1L sample, and to 7.2 × 10^–5^ S cm^–1^ for the KNN‐NZSP CPE‐2L sample, further to 6.0 × 10^–5^ S cm^–1^ for the KNN‐NZSP CPE‐3L sample, but still comparable among most reported CPEs.^[^ ^7^, ^15^, ^17^, ^18^, ^32^, ^33^ ^]^ Due to the effective ion‐migration pathways contributed by polymer wrappings, the KNN coatings do not strongly affect the Na^+^ conduction of the KNN‐NZSP CPEs. Furthermore, the electronic conductivity of the CPEs was measured via the potentiostatic polarization tests. In Figure 3b, the electronic conductivity of all the KNN‐engineered CPEs is less than 4 × 10^–9^ S cm^–1^ at room temperature, indicating that KNN‐NZSP CPEs remain electronically insulating.

As another requisite parameter, the electrochemical window of the CPEs was measured at room temperature. The KNN‐NZSP CPEs exhibit higher stability of about 5.0 ‐ 5.2 V (versus Na/Na^+^) than the non‐engineered NZSP CPE (4.8 V) in the anodic scanning (Figure 3(c1)). In Figure 3(c2), the cathodic stability is extended from 1.2 V for the NZSP CPE to 0.7‐0.9 V for the KNN‐NZSP CPEs. An electrochemical reaction between the CPE and Na electrode is hypothesized to occur during the cathodic scanning,^[^ ^4^, ^32^ ^]^ especially in the NZSP CPE. As further verified in Figure 3(c3) and Figure S3, Supporting Information, no Na dissolution current peak could be observed in the NZSP CPE when potential was swept from the open circuit potential (OCP) to −1.0 V, and back from −1.0 V to 6.0 V. Due to the different potentials between Na anode and CPEs, accumulated electron layer would form at the interface of the anode. During the cathodic scanning of the NZSP CPE, the deposited Na^+^ might be fully consumed in the combination with the interfacial accumulated electrons, and further react with the polymer electrolyte inside the CPE. Thus, no dissolution current could be observed in the following anodic scanning. In contrast, the dissolution current peaks are clearly observed in the KNN‐NZSP CPEs at around 0 V, revealing that KNN improves the compatibility of CPE against the Na metal. As the interfacial accumulated electrons could be attenuated by the oriented KNN polarizations, the consumption of the deposited Na^+^ would be suppressed, leaving some Na^+^ to be dissolved in the following anodic scanning. In short, ferroelectric engineering enhances the electrochemical stability of the CPEs, and thus the KNN‐NZSP CPEs with widened electrochemical windows could satisfy the requirements of most sodium batteries.

**Figure 4:**
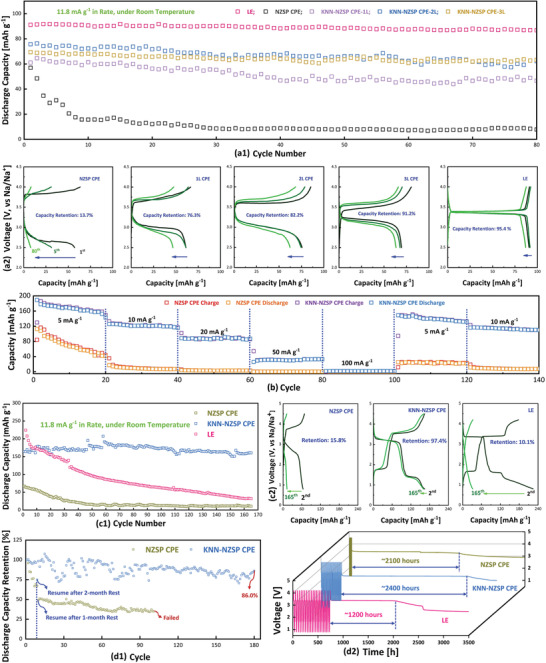
Electrochemical performance measured at room temperature: a1) Long‐term cycling performance and a2) charge/discharge profiles of the NVP | CPE | Na cells in comparison with the benchmark NVP | LE | Na cell, tested under a potential range of 2.5 ‐4.0 V; b) rate capability of the non/KNN‐engineered NVP | CPE | Na cells in a potential range of 0.8 ‐4.5 V; c1) long‐term cycling performance, c2) charge/discharge profiles of the NVP | electrolyte | Na cells, tested under extended potential range (0.8 ‐4.5 V for the CPE cells and 0.8‐ 4.2 V for the LE cell); d1) discharge capacity retention of the suspended NVP | CPE | Na cells, d2) stability comparison among the NVP | electrolyte | Na cells under full‐charged state.

Prototypes of the all‐solid‐state Na metal batteries with the non/KNN‐engineered CPEs were prepared, and the electrochemical performances of all cells were assessed at room temperature. A cell using NVP of similar mass loading and liquid electrolyte (LE, 1.0 M NaClO_4_ in EC/DEC = 1:1 vol% with 2% FEC) was prepared as the benchmark for comparison. **Figure** **4**a shows the capacities and cyclabilities of all cells measured in the potential range between 2.5 and 4.0 V at a specific current density of 11.8 mA g^–1^. The non‐engineered cell delivers a discharge capacity of 57.0 mAh g^–1^ at the 1^st^ cycle, and 31.1 mAh g^–1^ at the 5th cycle, but its capacity quickly drops to 12.2 mAh g^–1^ after 20 cycles and maintains the low capacity till the 80^th^ cycle with a capacity retention of only 13.7%. The severe capacity decay could be caused by the electrolyte‐electrode interfacial incompatibility and the giant interfacial resistance. As shown in Figure 4(a2), the NVP | NZSP CPE | Na cell shows a large and ever‐increased potential polarization during cycling. Compared to the non‐engineered cell, the KNN‐engineered cells demonstrate improved cyclic performance even though the KNN‐engineered CPE itself possesses slightly lower ionic conductivity. The NVP | KNN‐NZSP CPE‐1L | Na cell delivers an initial discharge capacity of 61.1 mAh g^–1^ and a retention of 76.3% after 80 cycles (Figure 4(a1)). It is interesting to note that with increased KNN thickness, namely the number of layers, the NVP | KNN‐NZSP CPE‐2L | Na cell shows improved initial discharge capacity of 75.7 mAh g^–1^ and enhanced retention of 82.2% over 80 cycles. Though there is a slight decrease in capacity but still higher than that of the 1L cell. The NVP | KNN‐NZSP CPE‐3L | Na cell delivers an initial discharge capacity of 69.4 mAh g^–1^ and further enhanced capacity retention of 91.2% after 80 cycles, which is very close to the benchmark retention of 95.4% in the LE cell. Furthermore, greatly reduced potential polarization is observed for the KNN‐NZSP CPE cells than the NZSP CPE cell (Figure 4(a2)). After cycling, the impedance of the above CPE cells was measured (Figure S4, Supporting Information), and much lower impedances for the KNN‐NZSP CPE cells were observed. In other words, the interfacial ferroelectric engineering greatly enhances the Na^+^ conduction within the whole all‐solid‐state cells, even though it slightly decreases the ionic conductivity of the CPE itself.

As known, the cathode NVP could provide additional capacity if it can transfer 3 electrons by (de)intercalating all three Na ions in an extended potential range. Considering the wide electrochemical window of the newly developed CPEs, we further measured the all‐solid‐state NVP | CPE | Na cells in the potential range between 0.8 and 4.5 V at room temperature. To avoid the high‐voltage decomposition of the LE, the electrochemical performance of the NVP | LE | Na cell was measured between 0.8 V and 4.2 V. Figure 4b shows the rate performance of the NVP | CPE with/without KNN | Na cells. Discharge capacity of 142.9, 116.0, 87.3, 33.2 and 1.8 mAh g^–1^ are obtained in the KNN‐engineered cell after 20 cycles, at the current density of 5, 10, 20, 50, and 100 mA g^–1^, respectively, superior over the capacity of 41.3, 7.6, 3.2, 0.4 and 0.02 mAh g^–1^ in the non‐engineered cell. When the current density reverts to 5 mA g^–1^ and 10 mA g^–1^, reversible capacity of 131.5 mAh g^–1^ and 110.1 mAh g^–1^ are achieved in the KNN‐engineered cell after 20 cycles, whereas 21.8 mAh g^–1^ and 7.7 mAh g^–1^ are obtained in the non‐engineered cell. Moreover, the long‐term cycling performance of the NVP | CPE | Na cells has been investigated and compared with the benchmark NVP | LE | Na cell, in the extended potential range (Figure 4c and Figure S5, Supporting Information). Excellent reversible capacity of 160.3 mAh g^–1^ with the retention of 97.4% is demonstrated in the NVP | KNN‐NZSP CPE | Na cell after 165 cycles, much higher than the non‐engineered NZSP CPE cell and even better than the benchmark LE cell. Besides, the KNN‐engineered cell only shows small and almost non‐increasing potential polarization over 165 full charge/discharge cycles. Considering the narrow electrochemical window of the conventional LE, the obvious capacity fading in the LE cell should be caused by the degradation of LE under the extended potential range, especially at low potential.^[^ ^34^ ^]^ On the contrary, the all‐solid‐state Na metal cell with the KNN‐NZSP CPE performs well under the tested wide potential range.

**Table** **1** summarizes the cyclic performance of the reported solid‐state Na metal cells using PEO‐based electrolytes. Small capacity retentions have been normally reported, especially at room temperature. At elevated temperatures, the electrolyte‐electrode interfacial resistance can be reduced to some extent, hence improving initial capacity and cycling retention. In the present work, the electrolyte‐electrode interfaces are improved via ferroelectric engineering, and record‐high retention has been achieved in our all‐solid‐state Na metal cell at room temperature.

**Table 1:** Cyclic performance of the solid‐state Na metal cells using PEO‐based electrolytes

Battery cell	Initial/theoretical discharge capacity [mAh g^–1^]	Cycle number	Current density [mA g^–1^]	Operating temperature [°C]	Discharge capacity retention [%]	Ref.
Na_0.44_Mn_0.67_Ti_0.33_O_2_ | Na_3_Zr_2_Si_2_PO_12_‐PEO (NaTFSI) | Na	96.2/120	350	10	RT	38.5	^[^ ^18^ ^]^
Y‐doped Na_2_ZrO_3_ | Emim FSI‐SiO_2_‐PEO (NaClO_4_) | Na	90.5/289	100	14.45	RT	51.0	^[^ ^17^ ^]^
	212.5/289	100	144.5	60	29.5	
Na_x_V_2_O_5_ | POSS‐PEG (NaClO_4_) | Na	305/>360	50	15	80	40.3	^[^ ^12^ ^]^
	264/>360	50	30	80	38.5	
	152/>360	50	60	80	48.7	
NaTi_2_(PO_4_)_3_ | HMOP‐PEO (NaTFSI) | Na	115/133	125	66.5	65	60.9	^[^ ^35^ ^]^
NaCu_1/9_Ni_2/9_Fe_1/3_Mn_1/3_O_2_ | NaFNFSI/PEO | Na	105/120	145	120	80	70.0	^[^ ^36^ ^]^
Na_3_V_2_(PO_4_)_3_ | PEO (NaClO_4_) | Na	93/118	70	11.8	60	28.9	^[^ ^37^ ^]^
Na_3_V_2_(PO_4_)_3_ | Pyr_13_FSI‐PEO (NaClO_4_) | Na	98/118	70	11.8	60	86.7	
Na_2_MnFe(CN)_6_ | Na_3_Zr_2_Si_2_PO_12_‐PEO (NaClO_4_) | Na	109.3/171	300	85.5	60	83.0	^[^ ^15^ ^]^
Na_3_V_2_(PO_4_)_3_ | Na_3_Zr_2_Si_2_PO_12_‐PEO (NaFSI) | Na	102/118	120	11.8	80	76.5	^[^ ^7^ ^]^
Na_3_V_2_(PO_4_)_3_ | Na_3.4_Zr_1.8_Mg_0.2_Si_2_PO_12_‐PEO (NaFSI) | Na	106.1/118	120	11.8	80	94.2	
Na_3_V_1.85_Fe_0.15_(PO_4_)_3_ | KNN‐Na_3_Zr_2_Si_2_PO_12_‐PEO (NaTFSI) | Na	164.6/177	165	11.8	RT	97.4	This work

More attractively, the present ferroelectric‐engineered cell demonstrates excellent stability even after long‐time aging. Due to the COVID‐19, the NVP | KNN‐NZSP CPE | Na cell has been suspended for 2 months during the charge/discharge cycling, but the cell could still perform well after resumption. Figure 4(d1) and Figure S6, Supporting Information display its cyclic capacity of ≈155 mAh g^–1^ before suspension, and this high discharge capacity quickly recovers after resumption. After 180 full charge/discharge cycles, a discharge capacity retention of 86.0% is achieved in the engineered cell. On the contrary, the previous capacity could not be recovered in the non‐engineered cell after resumption, although the cell has only been suspended for 1 month. The non‐engineered NVP | NZSP CPE | Na cell even fails quickly after resumption and 102 cycles. In Figure 4(d2), the stability of the NVP | CPE with/without KNN | Na cells was further evaluated, in comparison with the benchmark LE cell. As shown, all the cells have been charged/discharged 20 cycles followed by being fully charged and long‐term aged, and the open‐circuit potential of each cell is recorded. The potential keeps stable for about 2400 h in the KNN‐engineered cell, whereas it drops after about 2100 h in the non‐engineered cell and about 1200 hours in the LE cell. Obviously, the KNN‐engineered all‐solid‐state Na metal cell demonstrates outstanding stability in its full‐charged state.

**Figure 5:**
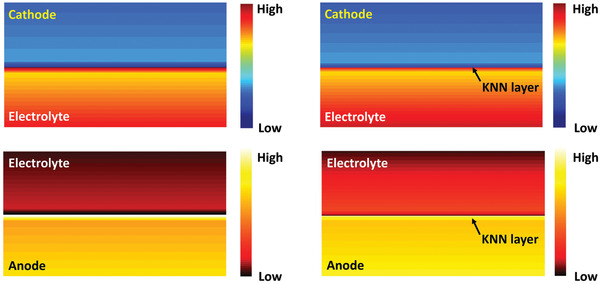
Simulated Na‐ion concentration at cathode‐electrolyte and anode‐electrolyte interfaces with/without KNN layer (the arrow indicates the position of KNN layers).

To better understand how the ferroelectric engineering influences the behavior of Na ions at the electrolyte‐electrode interfaces, a simulation model was developed (for more details, refer to Supporting Information). **Figure** **5** shows the calculated Na‐ion distribution at the electrolyte‐electrode interfaces with/without ferroelectric engineering. To make the interfacial concentration contrast clearer, the cathode‐electrolyte and anode‐electrolyte interfaces are separated in different images. The interfacial ion distribution is obviously abnormal in the non‐engineered cell. On the cathode‐electrolyte side, Na^+^‐accumulated and Na^+^‐deficient layers form at the interfacial electrolyte and cathode, separately. To maintain the equilibrium, a Na^+^‐ moderate layer appear close to the Na^+^‐accumulated layer within the interim electrolyte. On the anode‐electrolyte side, similar ion distributions are observed. The above‐mentioned space‐charge layers would all hinder the interfacial ion conduction. However, the oriented ferroelectric polarizations can effectively attenuate the space‐charge layers at the electrolyte‐ferroelectric‐electrode interfaces. In Figure S7, Supporting Information, the ferroelectric‐induced electric field weakens the sharp potential drop at the cathode/anode‐electrolyte interfaces. Thus, weakened interfacial ion changes are obtained at the electrolyte‐ferroelectric‐cathode/anode interfaces of the KNN‐engineered cell.

**Figure 6:**
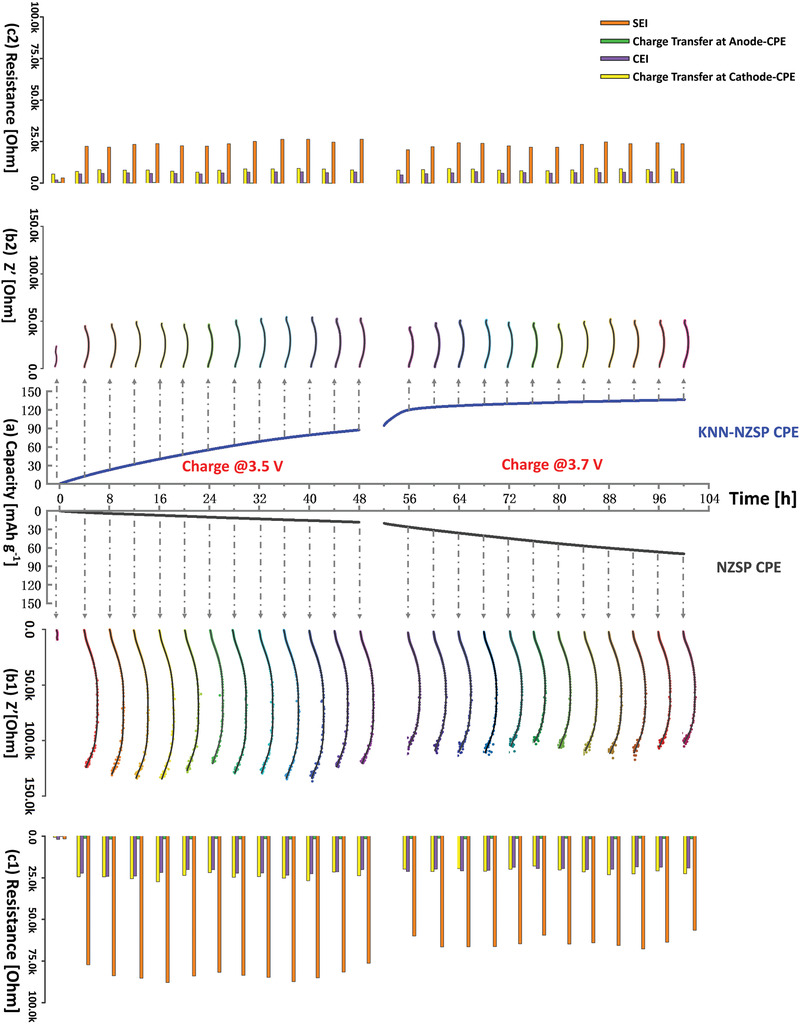
a) Capacity in the non/KNN‐engineered NVP | CPE | Na cells, under 3.5 V and 3.7 V charging; Impedance in b1) the non‐engineered and b2) KNN‐engineered cells before and during charging; Fitted SEI, CEI, cathode‐CPE charge‐transfer and anode‐CPE charge‐transfer resistances in c1) the non‐engineered and c2) KNN‐engineered cells. All measurements are conducted at room temperature.

To verify the reliability of the simulation and identify whether the ferroelectric engineering can enhance the ion conduction across the electrolyte‐electrode interfaces, a constant‐voltage‐charging measurement was designed to monitor the changes of impedance in the non/ferroelectric‐engineered NVP | CPE | Na cells. **Figure** **6** and Figure S8, Supporting Information show the impedance of each cell in‐situ recorded at a 4 h interval during the long‐term charging. Before charging, the impedance of the as‐prepared cells was measured, and the NZSP CPE cell shows smaller total resistance than that of the KNN‐NZSP CPE cell. During charging, the total resistance increases for both cells, but especially for the NZSP CPE cell, that increases more than one order of magnitude. By fitting the impedance plots, four individual interfacial components could be achieved, namely, SEI resistance, CEI (cathode electrolyte interphase) resistance, charge‐transfer resistance at anode‐electrolyte interface, and charge‐transfer resistance at cathode‐electrolyte interface. As observed, the SEI resistance is the major cause hindering the anode‐CPE interfacial ion conducting. The SEI resistance in the NVP | NZSP CPE | Na cell is 76.4 ‐ 88.1 kΩ under 3.5 V‐charging and 56.6 ‐ 68.0 kΩ under 3.7 V‐charging. Besides that, the large CEI resistance (18.7 ‐ 24.1 kΩ) and the cathode‐CPE charge transfer resistance (18.3 ‐ 27.6 kΩ) hinder the ion migration across the cathode‐CPE interface, resulting in the low capacity of 69.9 mAh g^–1^ in the non‐engineered NZSP CPE cell during the 100 h‐charging. On the contrary, the ferroelectric‐engineered cell demonstrates its outstanding capability in reducing the interfacial resistances, and therefore the NVP | KNN‐NZSP CPE | Na cell can quickly reach its full‐charged state. The SEI and cathode‐CPE charge transfer resistances of the KNN‐NZSP CPE cell are less than one‐third of that of the NZSP CPE cell, and the CEI resistance of the KNN‐engineered cell is about a quarter of that in the non‐engineered cell during charging. Notably, the anode‐CPE charge transfer resistance in the KNN‐NZSP CPE cell shows negligible changes before and under charging (keeps at about 0.4 kΩ), whereas the resistance increases nine‐fold in the NZSP CPE cell (from 0.2 to 1.8 kΩ).

Simulation predicts that the cell without ferroelectric engineering will form thick space‐charge layers at the interfacial electrolyte. These space charges hinder the interfacial ion transfer and it is therefore suggested to be the reason for the large cathode/anode‐CPE charge transfer resistances in the NZSP CPE cell. Furthermore, the calculated Na^+^ distribution gap at the interfacial cathode (Figure 5) might be the reason for the measured CEI resistance in the NZSP CPE cell. On the other hand, the accumulated electrons at the interfacial anode will continuously combine with the mobile Na^+^ and react with the polymer electrolyte, especially in the NZSP CPE cell. Consequently, huge SEI resistances are always observed in the NZSP CPE cell under high‐voltage charging. Contributed by the ferroelectric layers, the cathode/anode‐electrolyte interfacial space charges are greatly attenuated, and dramatically reduced cathode/anode‐CPE charge transfer resistances and CEI resistances are therefore demonstrated in the KNN‐NZSP CPE cell. Along with the weakened electron accumulation at the interfacial anode, the growth of the oxide SEI is also suppressed and small SEI resistances are maintained in the KNN‐NZSP CPE cell. As shown in Figure S10, Supporting Information, less oxide impurities are observed in the KNN‐NZSP CPE than the NZSP CPE, further evidencing the significance of the KNN‐engineering in suppressing the oxide SEIs.

## 3 Conclusion

We have developed an innovative approach, based on ferroelectric effect, to improve the electrolyte‐electrode compatibility and reduce the cathode/anode‐composite polymer electrolyte interfacial resistances in the all‐solid‐state sodium metal cells. Via the interfacial ferroelectric engineering, the SEI growth is effectively suppressed at the interface between the composite polymer electrolyte and the metallic Na, and the interfacial ion transportation has been enhanced at the electrolyte‐ferroelectric‐cathode/anode interfaces. An excellent discharge capacity of 160.3 mAh g^–1^, with 97.4% in retention, is achieved in the ferroelectric‐engineered all‐solid‐state Na metal cell after 165 cycles at room temperature. Furthermore, superior stability has been demonstrated in the ferroelectric engineered cells, and high discharge capacity retention of 86.0% is obtained in the engineered cell over 180 full charge/discharge cycles, even though the cell has been suspended for 2 months during cycling. It is therefore state‐of‐the‐art towards the development of high‐safety, high‐capacity and long‐cyclability solid‐state Na metal batteries. We believe this interfacial ferroelectric engineering strategy can be applied as a general approach to improve the cyclic performance and stability of various solid‐state batteries with polymer‐based electrolytes.

## 4 Experimental Section

### Preparation of the KNN‐NZSP CPEs

Porous NZSP ceramic frameworks^[^ ^18^ ^]^ and the KNN precursor solution^[^ ^38^ ^]^ were prepared as previously reported. The KNN solution was uniformly drop‐coated onto the NZSP frameworks. The coated frameworks were dried at 100 °C for 5 min, pyrolyzed at 550 °C for 10 min and annealed at 750 °C for 30 min in air. The weight ratio of KNN:NZSP was 1.3:100 in the annealed KNN‐NZSP 1L framework, 2.6:100 in the annealed KNN‐NZSP 2L framework, and 3.7:100 in the annealed KNN‐NZSP 3L framework. PEO (NaTFSI) filler was synthesized from PEO (Mv 600000, Sigma Aldrich) and NaTFSI (97 wt%, Sigma Aldrich) with the EO:Na molar ratio fixed at 12:1. Then, the KNN‐NZSP CPEs were fabricated by vacuum infiltrating^[^ ^39^ ^]^ the melted PEO (NaTFSI) into the pores of KNN‐NZSP frameworks. The polymer filling process was repeated until both sides of the KNN‐NZSP framework were wetted by the melted polymer. The soft KNN‐NZSP CPEs were further pressed into thin membrane with desired size (300 ≈ 500 µm in thickness).

### Characterization

The morphology of the KNN‐NZSP frameworks and the CPEs were characterized by the field emission scanning electron microscopy (FESEM, HITACHI S‐4300) and transmission electron microscopy (TEM, Tecnai G2 F30). The crystal structures of the frameworks and the CPEs were examined by X‐ray diffraction (XRD, Shimadzu XRD‐6000). Raman spectroscopy of the KNN‐NZSP framework was recorded using Raman Imaging Microscopy (Renishaw, inVia Raman microscope). Thermal behaviors of the CPEs were identified using a thermogravimetric‐differential thermal analysis apparatus (TG‐DTA, Shimadzu, DTG‐60H) with a heating rate of 10 °C min^–1^ in air. PFM was conducted on the KNN‐NZSP frameworks via a commercial SPM system (MFP‐3D, Asylum research, Oxford Instruments, CA, USA), using the Pt coated Si probes (AC 240 PP, OPUS, Bulgaria). Electrochemical impedance spectroscopy of the CPEs and Na metal cells were measured by a Solartron system (1260 + 1287), with an AC voltage of 10 mV. The ionic conductivity of the CPEs was calculated according to the equation: *σ* = *t*/RA, where t is the thickness of CPE, A is the surface area of CPE, and R is the measured resistance from impedance spectroscopy. Electronic conductivity of CPEs was measured through the potentiostatic test. The electrochemical window of the CPEs was evaluated via the linear cyclic voltammetry in a coin cell, with the metallic Na as the reference electrode and stainless steel as the working electrode.

### Electrochemical Measurements of the Battery Cells

NVP powders were synthesized as previously reported.^[^ ^40^ ^]^ Then, NVP powders, NZSP powders, super P and polyvinylidene fluoride were dissolved in *N*‐methyl‐2‐pyrrolidone with a weight ratio of 5:2:2:1 to prepare the cathode slurry. The addition of NZSP was to increase the ionic conductivity of the cathode. The cathodes were finally prepared by casting the slurry onto aluminum foil and vacuum drying at 120 °C overnight. The mass loading of active material was 0.9 ‐ 1.1 mg cm^–2^. All cells were assembled in the glovebox (O_2_ < 1 ppm and H_2_O < 1 ppm), via cold‐pressing the cathode, electrolyte, and metallic Na into coin cells. The rate performance of the cells was measured using the MACCOR system (SERIES‐4000). All the other electrochemical performances were tested using the LAND battery testing system.

## Conflict of Interest

The authors declare no conflict of interest.

## Supporting information

Supporting Information

## Data Availability

The data that support the findings of this study are available from the corresponding author upon reasonable request.

## References

[1] N. Yabuuchi , K. Kubota , M. Dahbi , S. Komaba , Chem. Rev. 2014, 114, 11636. 25390643 10.1021/cr500192f

[2] J. B. Goodenough , Y. Kim , Chem. Mater. 2010, 22, 587.

[3] Y. Wang , S. Song , C. Xu , N. Hu , J. Molenda , L. Lu , Nano Mater. Sci. 2019, 1, 91.

[4] F. Colò , F. Bella , J. R. Nair , M. Destro , C. Gerbaldi , Electrochim. Acta 2015, 174, 185.

[5] Y. L. Ni'mah , M. Y. Cheng , J. H. Cheng , J. Rick , B. J. Hwang , J. Power Sources 2015, 278, 375.

[6] X. Qi , Q. Ma , L. Liu , Y. S. Hu , H. Li , Z. Zhou , X. Huang , L. Chen , ChemElectroChem 2016, 3, 1741.

[7] Z. Zhang , Q. Zhang , C. Ren , F. Luo , Q. Ma , Y. S. Hu , Z. Zhou , H. Li , X. Huang , L. Chen , J. Mater. Chem. A 2016, 4, 15823.

[8] J. Mindemark , R. Mogensen , M. J. Smith , M. M. Silva , D. Brandell , Electrochem. Commun. 2017, 77, 58.

[9] C. Wang , Y. Yang , X. Liu , H. Zhong , H. Xu , Z. Xu , H. Shao , F. Ding , ACS Appl. Mater. Interfaces 2017, 9, 13694. 28334524 10.1021/acsami.7b00336

[10] L. Chen , Y. Li , S. P. Li , L. Z. Fan , C. W. Nan , J. B. Goodenough , Nano Energy 2018, 46, 176.

[11] S. Chen , F. Feng , Y. Yin , H. Che , X. Z. Liao , Z. F. Ma , J. Power Sources 2018, 399, 363.

[12] Y. Zheng , Q. Pan , M. Clites , B. W. Byles , E. Pomerantseva , C. Y. Li , Adv. Energy Mater. 2018, 8, 1801885.

[13] L. Liu , X. Qi , S. Yin , Q. Zhang , X. Liu , L. Suo , H. Li , L. Chen , Y. S. Hu , ACS Energy Lett. 2019, 4, 1650.

[14] C. Sångeland , R. Younesi , J. Mindemark , D. Brandell , Energy Storage Mater. 2019, 19, 31.

[15] X. Yu , L. Xue , J. B. Goodenough , A. Manthiram , ACS Mater. Lett. 2019, 1, 132.

[16] Q. Zhang , Y. Lu , H. Yu , G. Yang , Q. Liu , Z. Wang , L. Chen , Y. S. Hu , J. Electrochem. Soc. 2020, 167, 070523.

[17] S. Song , M. Kotobuki , F. Zheng , C. Xu , S. V. Savilov , N. Hu , L. Lu , Y. Wang , W. D. Z. Li , J. Mater. Chem. A 2017, 5, 6424.

[18] Y. Wang , Z. Wang , J. Sun , F. Zheng , M. Kotobuki , T. Wu , K. Zeng , L. Lu , J. Power Sources 2020, 454, 227949.

[19] A. C. Luntz , J. Voss , K. Reuter , J. Phys. Chem. Lett. 2015, 6, 4599. 26551954 10.1021/acs.jpclett.5b02352

[20] N. Ohta , K. Takada , L. Zhang , R. Ma , M. Osada , T. Sasaki , Adv. Mater. 2006, 18, 2226.

[21] C. Yada , A. Ohmori , K. Ide , H. Yamasaki , T. Kato , T. Saito , F. Sagane , Y. Iriyama , Adv. Energy Mater. 2014, 4, 1301416.

[22] Y. Shen , Y. Zhang , S. Han , J. Wang , Z. Peng , L. Chen , Joule 2018, 2, 1674.

[23] T. Famprikis , P. Canepa , J. A. Dawson , M. S. Islam , C. Masquelier , Nat. Mater. 2019, 18, 1278. 31427742 10.1038/s41563-019-0431-3

[24] H. Yamada , K. Suzuki , K. Nishio , K. Takemoto , G. Isomichi , I. Moriguchi , Solid State Ionics 2014, 262, 879.

[25] Z. Cheng , M. Liu , S. Ganapathy , C. Li , Z. Li , X. Zhang , P. He , H. Zhou , M. Wagemaker , Joule 2020, 4, 1311.

[26] A. Mirsakiyeva , M. Ebadi , C. M. Araujo , D. Brandell , P. Broqvist , J. Kullgren , J. Phys. Chem. C 2019, 123, 22851.

[27] S. Kaboli , H. Demers , A. Paolella , A. Darwiche , M. Dontigny , D. Clément , A. Guerfi , M. L. Trudeau , J. B. Goodenough , K. Zaghib , Nano Lett. 2020, 20, 1607. 32017575 10.1021/acs.nanolett.9b04452

[28] J. Haruyama , K. Sodeyama , L. Han , K. Takada , Y. Tateyama , Chem. Mater. 2014, 26, 4248.

[29] Y. Seino , T. Ota , K. Takada , J. Power Sources 2011, 196, 6488.

[30] K. Takada , N. Ohta , L. Zhang , K. Fukuda , I. Sakaguchi , R. Ma , M. Osada , T. Sasaki , Solid State Ionics 2008, 179, 1333.

[31] S. Jesse , A. P. Baddorf , S. V. Kalinin , Appl. Phys. Lett. 2006, 88, 062908.

[32] J. Serra Moreno , M. Armand , M. B. Berman , S. G. Greenbaum , B. Scrosati , S. Panero , J. Power Sources 2014, 248, 695.

[33] A. Boschin , P. Johansson , Electrochim. Acta 2015, 175, 124.

[34] A. Ponrouch , E. Marchante , M. Courty , J. M. Tarascon , M. R. Palacin , Energy Environ. Sci. 2012, 5, 8572.

[35] W. Zhou , H. Gao , J. B. Goodenough , Adv. Energy Mater. 2016, 6, 1501802.

[36] Q. Ma , J. Liu , X. Qi , X. Rong , Y. Shao , W. Feng , J. Nie , Y. S. Hu , H. Li , X. Huang , L. Chen , Z. Zhou , J. Mater. Chem. A 2017, 5, 7738.

[37] G. Chen , Y. Bai , Y. Gao , Z. Wang , K. Zhang , Q. Ni , F. Wu , H. Xu , C. Wu , ACS Appl. Mater. Interfaces 2019, 11, 43252. 31661238 10.1021/acsami.9b16294

[38] Y. Wang , K. Yao , M. Sharifzadeh Mirshekarloo , F. E. H. Tay , J. Am. Ceram. Soc. 2016, 99, 1631.

[39] J. Martin , C. Mijangos , Langmuir 2009, 25, 1181. 19138163 10.1021/la803127w

[40] T. Wu , J. Sun , M. Ke , C. Y. H. Lim , L. Lu , Mater. Des 2020, 186, 108287.

