# Supplementary material for: Ferroelectric Engineered Electrode‐Composite Polymer Electrolyte Interfaces for All‐Solid‐State Sodium Metal Battery
Source: Adv Sci (Weinh). 2022 Mar 6;9(13):2105849. doi: 10.1002/advs.202105849 (PMC9069353; doi:10.1002/advs.202105849)
Supplement: Supplementary file 1 — Supporting Information [file ADVS-9-2105849-s001.pdf]

ADVANCED SCIENCE
Open Access

Supporting Information

for *Adv. Sci.*, DOI 10.1002/advs.202105849

Ferroelectric Engineered Electrode-Composite Polymer Electrolyte Interfaces for All-Solid-State Sodium Metal Battery

Yumei Wang, Zhongting Wang, Feng Zheng, Jianguo Sun, Jin An Sam Oh, Tian Wu, Gongxuan Chen, Qing Huang, Masashi Kotobuki, Kaiyang Zeng and Li Lu**

Supporting Information

Ferroelectric Engineered Electrode-Composite Polymer Electrolyte Interfaces for All-Solid-State Sodium Metal Battery

Yumei Wang, Zhongting Wang, Feng Zheng, Jianguo Sun, Jin An Sam Oh, Tian Wu, Gongxuan Chen, Qing Huang, Masashi Kotobuki, Kaiyang Zeng, and Li Lu**

E-mail: yumei.wang@nusricq.cn, luli@nus.edu.sg

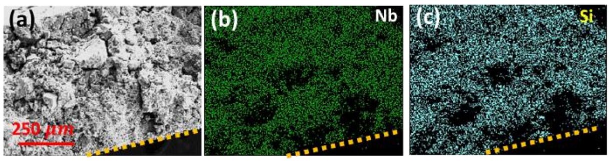

Figure S1. Cross-sectional (a) morphology, (b) Nb and (c) Si mapping of KNN-NZSP ceramic framework.

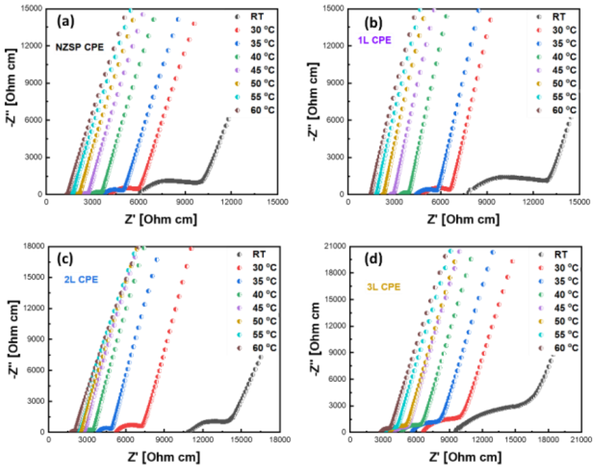

Figure S2. Temperature dependent electrochemical impedance spectroscopies of (a) NZSP CPE, (b) KNN-NZSP CPE-1L, (c) KNN-NZSP CPE-2L and (d) KNN-NZSP CPE-3L.

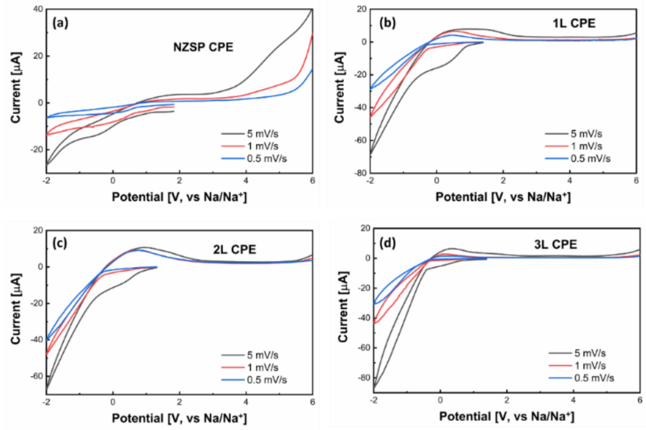

Figure S3. Current-potential curves of (a) non-engineered NZSP CPE, and (b~d) ferroelectric-engineered KNN-NZSP CPE-1L, 2L and 3L, measured at room temperature with various sweeping rates in the potential sequence from open circuit potential (OCP) to -2.0 V and back from -2.0 V to 6.0 V (vs. Na/Na^+).

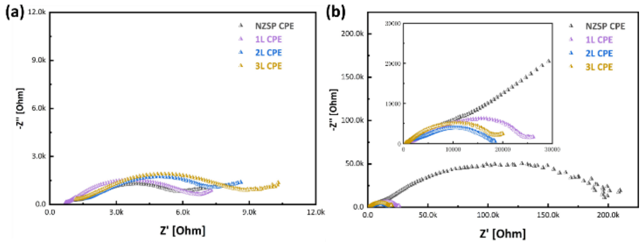

Figure S4. Electrochemical impedance spectroscopies of the NVP | CPE | Na cells with the NZSP CPE and the KNN-NZSP CPE-1L, 2L and 3L, (a) before and (b) after long-term cycling.

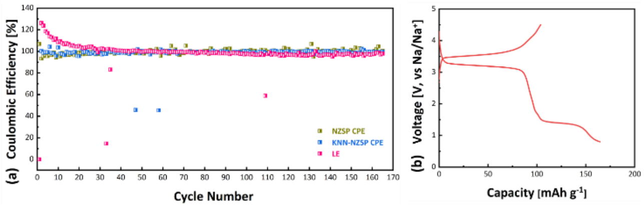

Figure S5. (a) Coulombic efficiency of the NVP | electrolyte | Na cells with LE, NZSP CPE and KNN-NZSP CPE in long-term cycling; (b) 1st galvanostatic charge-discharge profile of the NVP | KNN-NZSP CPE | Na cell. Considering the narrow electrochemical window of LE, the test voltage range is 0.8~4.2 V for the LE cell, and 0.8~4.5 V for the NZSP CPE cell and the KNN-NZSP CPE cell. All measurements were conducted at room temperature.

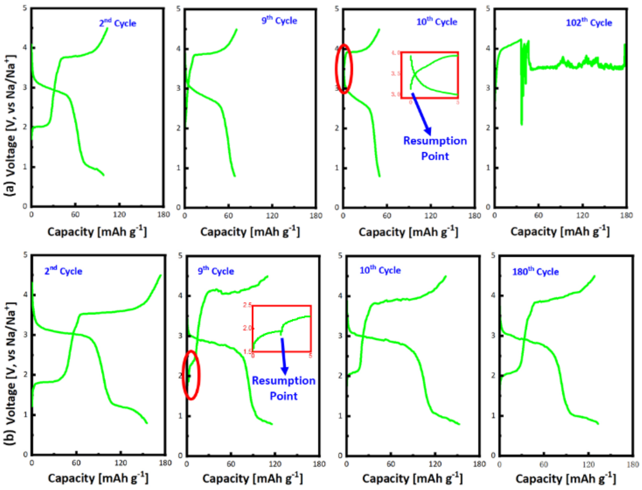

Figure S6. Charge-discharge profiles of the (a) NVP | NZSP CPE | Na cell and (b) NVP | KNN-NZSP CPE | Na cell before and after long-term suspension. All measurements were conducted at room temperature.

One dimensional finite element model is set up to investigate the influence of KNN engineering at the electrolyte-electrode interfaces. In the simulation model, only the electrode-electrolyte interfaces are studied, and the thicknesses of the interfacial cathode, anode and electrolyte are all set as 5 μm, while the thickness of KNN as 0.7 μm. Outside the interfaces, to simplify the model, the electric potential and ion concentration are all treated as constant. The current density *J* within the model can be expressed as:

$$J = -DF\frac{\partial c}{\partial x} \qquad \text{(non-engineered cell)}$$

$$J = -DF\frac{\partial c}{\partial x} + \frac{DF^2}{RT}cE \qquad \text{(KNN-engineered cell)}$$

where c is the molar ion concentration, *D* is effective diffusivity, *F* is Faraday constant, *R* is gas constant, *T* is temperature and *E* is the built-in electric filed generated by KNN.

By solving the above partial differential equations, the ion concentration distribution can be obtained, and then the electric potential can be calculated by ion concentration.

Here, the KNN-induced electric field, *E*, mainly depends on its polarizations. We treat the oriented KNN single domain as a pair of point charges, and one end with charge +q and the other one with charge -q. The displacement vector $\vec{d}$ can be regarded as negative to positive.

Then the dipole moment can be written as:

$$\vec{p} = q\vec{d}$$

Assuming the location of +q is $\vec{r_+}$ while the location of -q is $\vec{r_-}$, then $\vec{d} = \vec{r_+} - \vec{r_-}$.

The electric potential at location $\vec{r}$ can be expressed as:

$$u(\vec{r}) = \frac{q}{4\pi\varepsilon_0|\vec{r} - \vec{r_+}|} - \frac{q}{4\pi\varepsilon_0|\vec{r} - \vec{r_-}|}$$

Let $\vec{R}$ as the position vector relative to the mid-point $\frac{\vec{r_+}+\vec{r_-}}{2}$, and $\vec{e}$ is the corresponding unit vector:

$$\vec{R} = \vec{r} - \frac{\vec{r_+}+\vec{r_-}}{2}, \vec{e} = \frac{\vec{R}}{|R|},$$

By Taylor Expansion and omitting the terms of higher order, the potential can be expressed as:

$$u(\vec{R}) = \frac{\vec{p}\cdot\vec{e}}{4\pi\varepsilon_0|R|^2}$$

Then the electric field is the negative gradient of the potential, leading to

$$\vec{E}(\vec{R}) = \frac{3(\vec{p}\cdot\vec{R})\vec{R} - \vec{p}}{4\pi\varepsilon_0|R|^3}$$

The total electric field of KNN coating can be expressed as:

$$\vec{E}_{total} = \sum \vec{E_i}$$

Considering the small thickness of KNN, we take an acceptable E value of 4 $kV\ m^{-1}$ in the calculation, and the other parameters are: D_{anode}, $20 \times 10^{-10}\ m^2\ s^{-1}$; $D_{cathode}$, $5 \times 10^{-10}\ m^2\ s^{-1}$; $D_{electrolyte}$, $10 \times 10^{-10}\ m^2\ s^{-1}$; T, 300 K; J, 1 $mA\ cm^{-2}$.

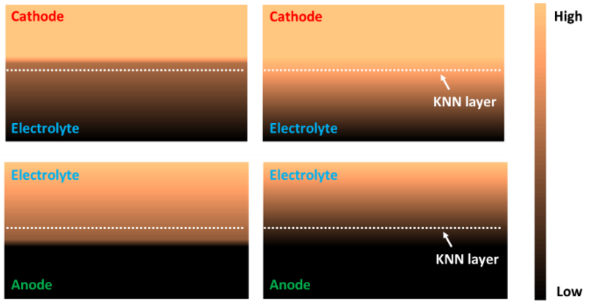

Figure S7. Simulated potential changes at the cathode-electrolyte and anode-electrolyte interfaces with/without KNN engineering (the arrow indicates the position of KNN layer, and the dash line indicates the electrolyte-electrode physical interface before charging).

After charging under 3.5 V and 3.7 V, the NVP | NZSP CPE | Na cell is further charged under 4.0 V to reach its full-charged state. Figure S8 shows the impedance changes of the cell under 4.0 V long-term charging. As a comparison, the NVP | KNN-NZSP CPE | Na cell is also charged under 4.0 V. However, to avoid over-charging, the KNN-NZSP CPE cell has been fully discharged before the 4.0 V-charging. The impedance plots of the NVP | CPE with/without KNN | Na cells in Figure 6 and Figure S8 are well fitted with the inserted equivalent circuits.

In the equivalent circuit, R1 represents the electronic resistance of the cell. (R2 || C1) correspond to the electrolyte resistance (the semicircle at high frequency, 10^6~10^5 Hz) and the estimated C1 is ~10^{-9} F. R3 and the parallel CPE1 (~10^{-6} F) correspond to the SEI resistance and its fitted semicircle is at low frequency with characteristic frequency of 10~100 Hz, whereas R4 || C2 (the semicircle at middle frequency whose characteristic frequency is 10^4~10^5 Hz) represents the charge transfer resistance at anode-electrolyte interface.[S1] The fitted (R3 || CPE1) and (R4 || C2) values agree well with the fitting results in the symmetric Na | CPE | Na cells (Figure S9). On the other hand, the elements (R5 || C3) and (R6 || C4) represent the CEI and cathode-electrolyte charge transfer resistances, respectively. The characteristic frequency of the fitted semicircle is ~10^2 Hz for the cathode-electrolyte charge transfer, and is ~10^3 Hz for the CEI component.[S2, S3]

As shown in Figure S8, even under 4.0 V-charging, much lower interfacial resistances (*i.e.*, SEI, CEI, cathode-electrolyte and anode-electrolyte charge transfer resistances) are obtained in the ferroelectric-engineered cell than the non-engineered cell. After the 4.0 V-charging, both cells have been aged for 1 month, and greatly increased interfacial resistances are clearly observed after aging, especially in the NZSP CPE cell. This is because the mobile Na^+ are continuously depleted with the SEI growth, and the new space-charge layers always form at the interfaces. Thanks to the ferroelectric-engineering, the oriented KNN polarizations could attenuate the interfacial space charges and suppress the SEI growth. Thus, well-controlled interfacial resistances have been achieved in the KNN-engineered cell even after charging and long-time aging.

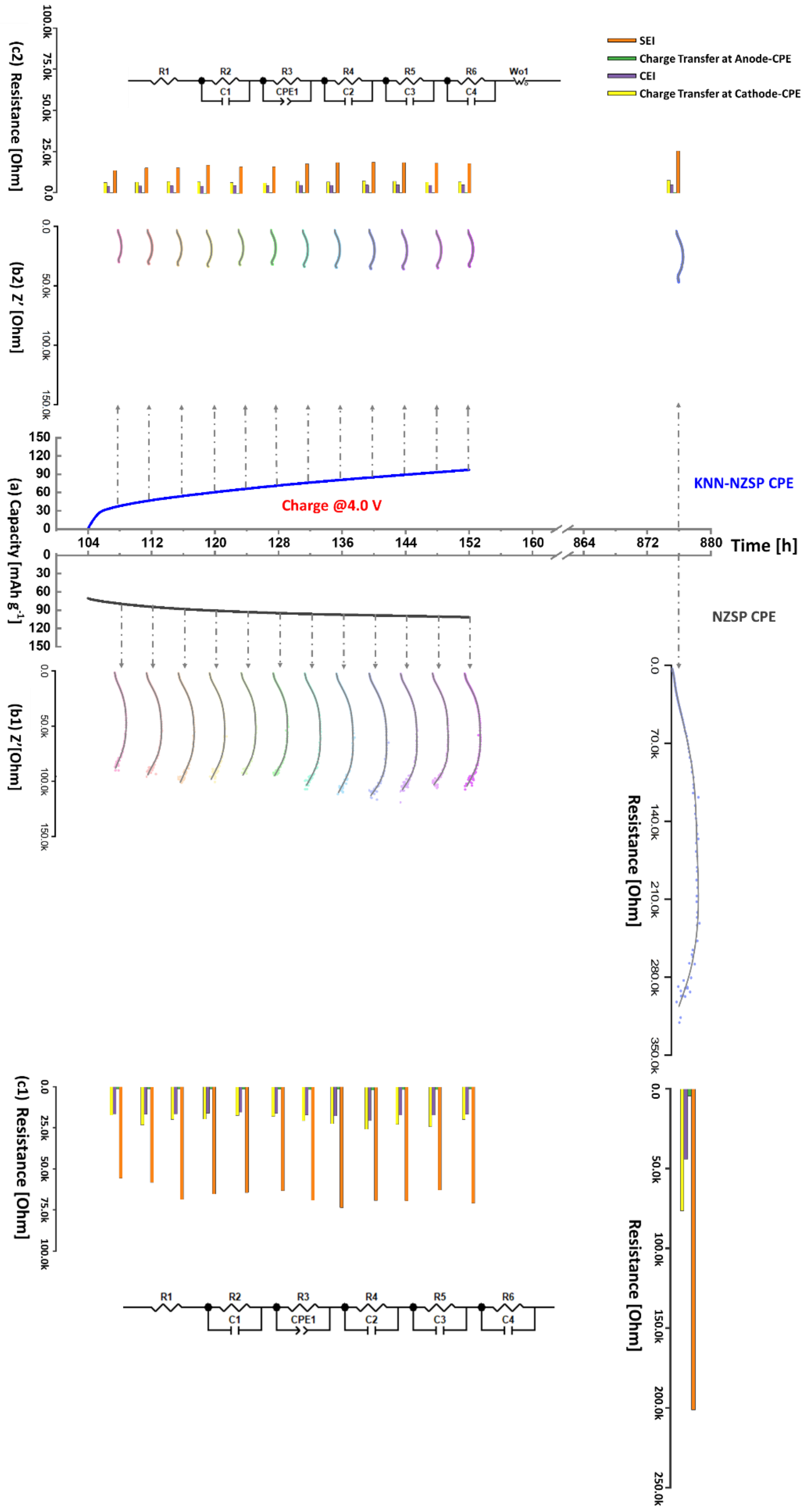

Figure S8. (a) Capacity in the non/KNN-engineered NVP | CPE | Na cells, under 4.0 V charging; Electrochemical impedance spectroscopy in (b1) the NZSP CPE and (b2) KNN-NZSP CPE cells during charging and after one-month aging; Fitted SEI, CEI, cathode-CPE charge-transfer and anode-CPE charge-transfer resistances in (c1) the NZSP CPE and (c2) KNN-NZSP CPE cells. Insets are the equivalent circuits for fitting the impedance plots. All measurements are conducted at room temperature.

In Figure S9, when Na | CPE | Na cells are under high-voltage charging, Na^+ migrates from positive Na (cathode) to negative Na (anode), and would combine with the accumulated electrons at the interfaces to transform into Na. The newly formed Na would react with the electrolyte, producing the oxide SEI/CEI at the anode/cathode-electrolyte interfaces. Thus, huge SEI and CEI resistances are demonstrated in the Na | NZSP CPE | Na cell, which empirically agrees with that in the NVP | CPE | Na cells, considering the individual specificity of the cells. However, the ferroelectric KNN presents its good capability in controlling the interfacial space charges, and it could also enhance the compatibility between CPEs and Na metal electrode. Therefore, greatly reduced interfacial resistances are achieved in the Na | KNN-NZSP CPE | Na cell.

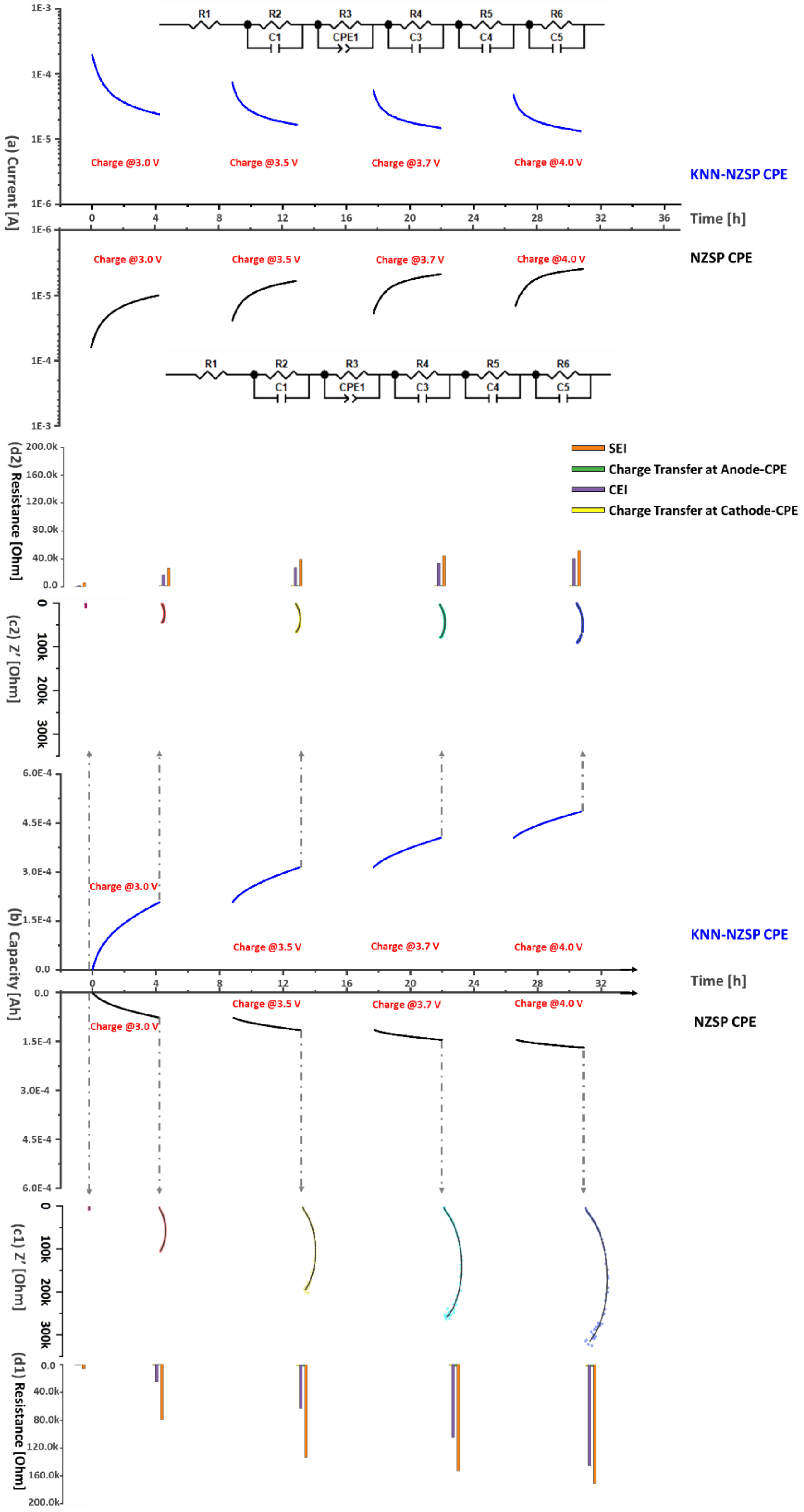

Figure S9. (a) Current and (b) Capacity of the Na | non/KNN-engineered CPE | Na symmetric cells under various voltage charging (Insets are the equivalent circuits for impedance fitting); (c) Electrochemical impedance spectroscopy and (d) the fitted SEI, CEI, cathode-CPE charge-transfer and anode-CPE charge-transfer resistances in the NZSP CPE and KNN-NZSP CPE cells before and under charging; All measurements are conducted at room temperature.

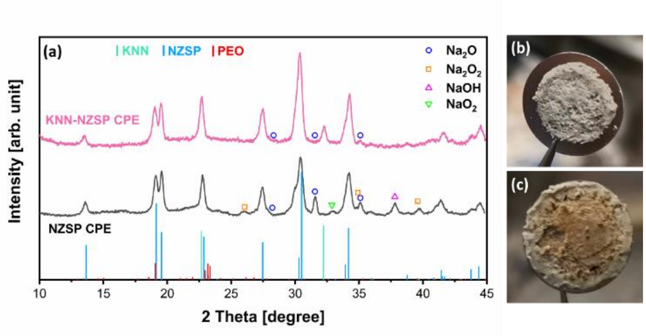

Figure S10. (a) XRD patterns of the KNN-NZSP CPE and NZSP CPE, that are disassembled from the NVP | CPE | Na cells after long-term charging and aging; (b) photograph of the KNN-NZSP CPE with top surface previously in contact with metallic Na; (c) photograph of the NZSP CPE with top surface previously in contact with metallic Na.

References

[S1] H. Kitaura, & H. Zhou, *Adv. Energy Mater.*, **2012**, 2, 889-894.

[S2] D. Aurbach, *J. Power Sources*, **2000**, 89, 206-218.

[S3] J. P. Schmidt, T. Chrobak, M. Ender, J. Illig, D. Klotz & E. Ivers-Tiffée, *J. Power Sources*, **2011**, 196, 5342-5348.
